# 3-(3-Chloro-2-hy­droxy­phen­yl)-1-phenyl-1*H*-pyrazole-4-carbaldehyde

**DOI:** 10.1107/S1600536811038025

**Published:** 2011-09-30

**Authors:** Pradeep Lokhande, Kamal Hasanzadeh, Hamid Khaledi, Hapipah Mohd Ali

**Affiliations:** a Department of Chemistry, University of Pune, Pune 411007, India; b Department of Chemistry, University of Malaya, 50603 Kuala Lumpur, Malaysia

## Abstract

In the title compound, C_16_H_11_ClN_2_O_2_, the pyrazole ring makes dihedral angles of 11.88 (13) and 22.33 (13)° with the 3-chloro-2-hy­droxy­benzene group and phenyl rings, respectively. The phenolic hy­droxy group forms an intra­molecular O—H⋯N hydrogen bond with the imine N atom of the pyrazole unit. The formyl group is virtually coplanar with the pyrazole ring [dihedral angle = 4.5 (19)°] and acts as an acceptor in an intra­molecular C—H⋯O hydrogen bond closing seven-membered ring. In the crystal, adjacent mol­ecules are linked through C—H⋯O hydrogen bonds into infinite chains along the *b* axis.

## Related literature

For structures of similar compounds, see: Jeyakanthan *et al.* (2001 ▶); Shanmuga Sundara Raj *et al.* (1999 ▶).
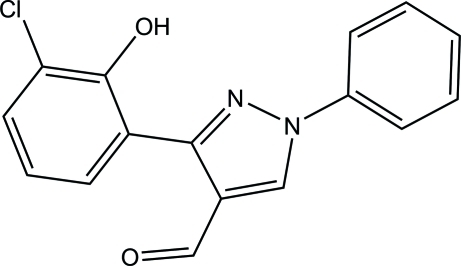


## Experimental

### 

#### Crystal data

C_16_H_11_ClN_2_O_2_
*M* *_r_* = 298.72
Orthorhombic, 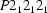

*a* = 3.8142 (1) Å
*b* = 15.9367 (3) Å
*c* = 21.4121 (5) Å
*V* = 1301.55 (5) Å^3^
*Z* = 4
Mo *K*α radiation
μ = 0.30 mm^−1^
*T* = 100 K
0.11 × 0.06 × 0.04 mm

#### Data collection

Bruker APEXII CCD diffractometer
Absorption correction: multi-scan (*SADABS*; Sheldrick, 1996 ▶) *T*_min_ = 0.968, *T*_max_ = 0.988
11133 measured reflections
2563 independent reflections
2195 reflections with *I* > 2σ(*I*)
*R*_int_ = 0.061

#### Refinement

*R*[*F* ^2^ > 2σ(*F* ^2^)] = 0.038
*wR*(*F* ^2^) = 0.075
*S* = 1.04
2563 reflections
223 parameters
Only H-atom coordinates refined
Δρ_max_ = 0.20 e Å^−3^
Δρ_min_ = −0.23 e Å^−3^
Absolute structure: Flack (1983 ▶), 1005 Friedel pairs
Flack parameter: −0.03 (7)

### 

Data collection: *APEX2* (Bruker, 2007 ▶); cell refinement: *SAINT* (Bruker, 2007 ▶); data reduction: *SAINT*; program(s) used to solve structure: *SHELXS97* (Sheldrick, 2008 ▶); program(s) used to refine structure: *SHELXL97* (Sheldrick, 2008 ▶); molecular graphics: *X-SEED* (Barbour, 2001 ▶); software used to prepare material for publication: ’*SHELXL97* and *publCIF* (Westrip, 2010 ▶)’.

## Supplementary Material

Crystal structure: contains datablock(s) I, global. DOI: 10.1107/S1600536811038025/gk2410sup1.cif

Structure factors: contains datablock(s) I. DOI: 10.1107/S1600536811038025/gk2410Isup2.hkl

Supplementary material file. DOI: 10.1107/S1600536811038025/gk2410Isup3.cml

Additional supplementary materials:  crystallographic information; 3D view; checkCIF report

## Figures and Tables

**Table 1:** Hydrogen-bond geometry (Å, °)

*D*—H⋯*A*	*D*—H	H⋯*A*	*D*⋯*A*	*D*—H⋯*A*
O1—H1⋯N2	0.79 (3)	1.89 (3)	2.585 (2)	147 (3)
C5—H5⋯O2	0.95 (2)	2.18 (2)	3.024 (3)	148 (2)
C10—H10⋯O1^i^	1.00 (3)	2.58 (3)	3.568 (3)	171 (2)

Symmetry code: (i) 
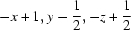
.

**Fig. 1.**
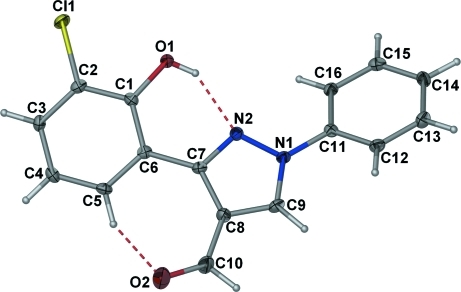
Molecular structure of the title compound with displacement ellipsoids at the 50% probability level. Hydrogen atoms are drawn as spheres of arbitrary radius. Intramolecular H-bonds are depicted as red dashed lines.

**Fig. 2.**
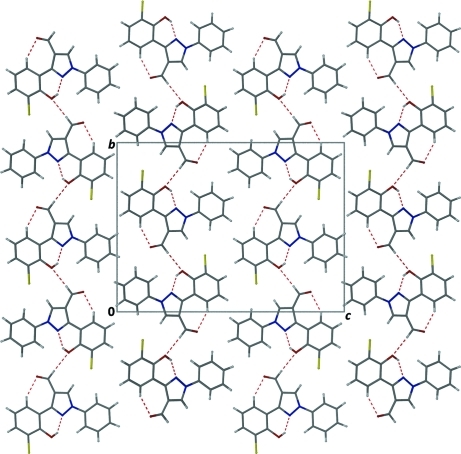
Packing view along the a axis showing hydrogen-bonded chains along the b axis. Hydrogen bonds are depicted as red dashed lines

## supplementary crystallographic information

### Comment

The title compound was synthesized through the action of Vilsmeier–Haack reagent (DMF/POCl_3_) on 3-chloro-2-hydroxyacetophenone phenylhydrazone. The compound contains three aromatic rings, the dihedral angles between them being 11.88 (13)° (pyrazole and phenol), 22.33 (13)° (pyrazole and phenyl) and 31.29 (12)° (phenyl and phenol). The phenol hydroxyl is hydrogen bonded to the pyrazole nitrogen, N2, and the formyl oxygen atom is directed towards the phenol ring to make an intramolecular C—H···O hydrogen bond with C5—H5. In contrary, in the crystal structures of the related compounds (Jeyakanthan *et al.*, 2001; Shanmuga Sundara Raj *et al.*, 1999) the formyl oxygen atoms are directed away from the phenol rings, being involved in intermolecular C—H···O hydrogen bonding. The crystal packing of the present compound exhibits infinite chains along the *b* axis formed by intermoleculoar C—H···O hydrogen bonds (Table 1).

### Experimental

A mixture of equivalent amounts (24 mmol) of 3-chloro-2-hydroxyacetophenone and phenyl hydrazine in methanol (40 ml) was refluxed for 2 h. The reaction mixture was then cooled to room temperature whereupon the condensation product, 3-chloro-2-hydroxy acetophenone phenylhydrazone, was seperated out with 92% yield. The hydrazone (2.6 g, 0.01 mol) was dissolved in DMF (15 ml) and then POCl_3_ (0.03 mol) was added dropwise at 0 *^o^*C. After the addition was complete, the reaction mixture was warmed to 60–70 *^o^*C and stirred for 2.5 h. The mixture was then poured onto crushed ice and neutralized by aqueous NaOH solution (10%). The precipitate was filtered, strongly washed with water and recrystallized from ethanol, yielding 85% of the pyrazole product (m.p. = 422-423 K). The needle shaped crystals of the compound were grown in a DMF solution at room temperature.

### Refinement

Hydrogen atoms were all located in a difference Fourier map and their positions refined with *U*_iso_(H) set to 1.2*U*_eq_(C) or 1.2*U*_eq_(O).

### Crystal data

**Table:**

C_16_H_11_ClN_2_O_2_	*F*(000) = 616
*M**_r_* = 298.72	*D*_x_ = 1.524 Mg m^−^^3^
Orthorhombic, *P*2_1_2_1_2_1_	Mo *K*α radiation, λ = 0.71073 Å
Hall symbol: P 2ac 2ab	Cell parameters from 1835 reflections
*a* = 3.8142 (1) Å	θ = 2.3–27.7°
*b* = 15.9367 (3) Å	µ = 0.30 mm^−^^1^
*c* = 21.4121 (5) Å	*T* = 100 K
*V* = 1301.55 (5) Å^3^	Needle, colorless
*Z* = 4	0.11 × 0.06 × 0.04 mm

### Data collection

**Table:**

Bruker APEXII CCD diffractometer	2563 independent reflections
Radiation source: fine-focus sealed tube	2195 reflections with *I* > 2σ(*I*)
graphite	*R*_int_ = 0.061
φ and ω scans	θ_max_ = 26.0°, θ_min_ = 2.3°
Absorption correction: multi-scan (*SADABS*; Sheldrick, 1996)	*h* = −4→4
*T*_min_ = 0.968, *T*_max_ = 0.988	*k* = −19→19
11133 measured reflections	*l* = −26→26

### Refinement

**Table:**

Refinement on *F*^2^	Secondary atom site location: difference Fourier map
Least-squares matrix: full	Hydrogen site location: inferred from neighbouring sites
*R*[*F*^2^ > 2σ(*F*^2^)] = 0.038	Only H-atom coordinates refined
*wR*(*F*^2^) = 0.075	*w* = 1/[σ^2^(*F*_o_^2^) + (0.0338*P*)^2^] where *P* = (*F*_o_^2^ + 2*F*_c_^2^)/3
*S* = 1.04	(Δ/σ)_max_ < 0.001
2563 reflections	Δρ_max_ = 0.20 e Å^−^^3^
223 parameters	Δρ_min_ = −0.23 e Å^−^^3^
0 restraints	Absolute structure: Flack (1983), 1005 Friedel pairs
Primary atom site location: structure-invariant direct methods	Flack parameter: −0.03 (7)

### Special details

**Table:**

Geometry. All e.s.d.'s (except the e.s.d. in the dihedral angle between two l.s. planes) are estimated using the full covariance matrix. The cell e.s.d.'s are taken into account individually in the estimation of e.s.d.'s in distances, angles and torsion angles; correlations between e.s.d.'s in cell parameters are only used when they are defined by crystal symmetry. An approximate (isotropic) treatment of cell e.s.d.'s is used for estimating e.s.d.'s involving l.s. planes.
Refinement. Refinement of *F*^2^ against ALL reflections. The weighted *R*-factor *wR* and goodness of fit *S* are based on *F*^2^, conventional *R*-factors *R* are based on *F*, with *F* set to zero for negative *F*^2^. The threshold expression of *F*^2^ > σ(*F*^2^) is used only for calculating *R*-factors(gt) *etc*. and is not relevant to the choice of reflections for refinement. *R*-factors based on *F*^2^ are statistically about twice as large as those based on *F*, and *R*- factors based on ALL data will be even larger.

### Fractional atomic coordinates and isotropic or equivalent isotropic displacement parameters (Å^2^)

**Table:**

	*x*	*y*	*z*	*U*_iso_*/*U*_eq_	
Cl1	−0.15696 (17)	0.83249 (4)	0.11291 (3)	0.02120 (16)	
O1	0.1795 (5)	0.74015 (10)	0.21221 (7)	0.0193 (4)	
H1	0.288 (8)	0.7150 (17)	0.2372 (12)	0.029*	
O2	0.2257 (7)	0.38231 (11)	0.14904 (10)	0.0523 (8)	
N1	0.6317 (6)	0.55861 (11)	0.30360 (8)	0.0146 (4)	
N2	0.4904 (5)	0.61122 (12)	0.26021 (8)	0.0152 (5)	
C1	0.1243 (7)	0.69086 (14)	0.16139 (10)	0.0146 (5)	
C2	−0.0338 (6)	0.72717 (14)	0.10952 (12)	0.0166 (5)	
C3	−0.0909 (6)	0.68296 (14)	0.05499 (11)	0.0170 (6)	
H3	−0.205 (7)	0.7091 (14)	0.0212 (11)	0.020*	
C4	0.0085 (7)	0.59966 (16)	0.05228 (11)	0.0189 (6)	
H4	−0.027 (6)	0.5695 (15)	0.0148 (11)	0.023*	
C5	0.1558 (7)	0.56110 (14)	0.10338 (10)	0.0169 (5)	
H5	0.217 (7)	0.5034 (14)	0.1021 (11)	0.020*	
C6	0.2166 (6)	0.60481 (14)	0.15903 (10)	0.0136 (5)	
C7	0.3790 (7)	0.56342 (13)	0.21291 (11)	0.0145 (5)	
C8	0.4516 (7)	0.47684 (15)	0.22672 (11)	0.0193 (6)	
C9	0.6108 (7)	0.47880 (15)	0.28431 (11)	0.0185 (6)	
H9	0.705 (7)	0.4337 (15)	0.3073 (11)	0.022*	
C10	0.3808 (9)	0.39580 (16)	0.19719 (13)	0.0340 (8)	
H10	0.478 (7)	0.3494 (17)	0.2230 (13)	0.041*	
C11	0.7764 (6)	0.59165 (14)	0.36014 (10)	0.0148 (6)	
C12	0.8074 (7)	0.54004 (15)	0.41212 (11)	0.0177 (5)	
H12	0.726 (7)	0.4860 (15)	0.4085 (10)	0.021*	
C13	0.9498 (7)	0.57231 (16)	0.46616 (12)	0.0205 (6)	
H13	0.959 (7)	0.5372 (16)	0.5017 (11)	0.025*	
C14	1.0616 (6)	0.65466 (16)	0.46938 (12)	0.0194 (6)	
H14	1.158 (7)	0.6750 (14)	0.5087 (11)	0.023*	
C15	1.0272 (7)	0.70584 (16)	0.41707 (11)	0.0192 (6)	
H15	1.114 (7)	0.7627 (15)	0.4189 (10)	0.023*	
C16	0.8840 (6)	0.67472 (15)	0.36244 (11)	0.0161 (5)	
H16	0.865 (7)	0.7101 (14)	0.3265 (11)	0.019*	

### Atomic displacement parameters (Å^2^)

**Table:**

	*U*^11^	*U*^22^	*U*^33^	*U*^12^	*U*^13^	*U*^23^
Cl1	0.0235 (3)	0.0144 (3)	0.0257 (3)	0.0028 (3)	−0.0039 (3)	0.0023 (3)
O1	0.0271 (10)	0.0136 (9)	0.0173 (9)	0.0022 (8)	−0.0054 (8)	−0.0012 (7)
O2	0.090 (2)	0.0165 (10)	0.0498 (13)	−0.0030 (11)	−0.0428 (14)	−0.0012 (9)
N1	0.0162 (11)	0.0122 (10)	0.0155 (10)	0.0009 (9)	0.0023 (10)	0.0013 (8)
N2	0.0157 (11)	0.0160 (11)	0.0138 (10)	0.0010 (9)	0.0018 (9)	0.0010 (8)
C1	0.0146 (13)	0.0148 (12)	0.0145 (12)	−0.0027 (10)	0.0019 (11)	−0.0013 (9)
C2	0.0133 (13)	0.0138 (12)	0.0226 (12)	−0.0001 (10)	0.0022 (11)	0.0026 (12)
C3	0.0168 (14)	0.0189 (14)	0.0153 (12)	−0.0024 (10)	−0.0030 (10)	0.0032 (10)
C4	0.0205 (15)	0.0216 (14)	0.0145 (12)	−0.0030 (11)	0.0004 (11)	−0.0029 (11)
C5	0.0187 (13)	0.0144 (12)	0.0177 (13)	0.0014 (12)	0.0039 (12)	−0.0032 (10)
C6	0.0116 (14)	0.0132 (12)	0.0159 (12)	−0.0020 (9)	0.0043 (10)	0.0023 (10)
C7	0.0122 (13)	0.0124 (12)	0.0188 (12)	−0.0005 (11)	0.0014 (11)	−0.0009 (9)
C8	0.0228 (16)	0.0160 (13)	0.0192 (13)	0.0003 (11)	−0.0040 (11)	−0.0013 (10)
C9	0.0187 (15)	0.0130 (12)	0.0238 (13)	0.0024 (11)	−0.0002 (12)	0.0034 (10)
C10	0.050 (2)	0.0159 (14)	0.0359 (17)	0.0009 (15)	−0.0215 (17)	−0.0009 (12)
C11	0.0114 (15)	0.0172 (13)	0.0159 (12)	0.0004 (10)	0.0021 (10)	−0.0019 (10)
C12	0.0168 (14)	0.0139 (12)	0.0224 (13)	0.0001 (11)	−0.0008 (11)	0.0002 (10)
C13	0.0211 (16)	0.0197 (14)	0.0208 (13)	0.0007 (11)	−0.0016 (11)	0.0046 (11)
C14	0.0170 (15)	0.0238 (15)	0.0174 (12)	0.0009 (11)	−0.0026 (11)	−0.0041 (11)
C15	0.0158 (14)	0.0153 (13)	0.0264 (14)	0.0006 (10)	0.0020 (11)	−0.0016 (11)
C16	0.0149 (13)	0.0177 (13)	0.0158 (11)	0.0031 (12)	0.0026 (11)	0.0011 (10)

### Geometric parameters (Å, °)

**Table:**

Cl1—C2	1.745 (2)	C6—C7	1.466 (3)
O1—C1	1.358 (3)	C7—C8	1.438 (3)
O1—H1	0.79 (3)	C8—C9	1.375 (3)
O2—C10	1.208 (3)	C8—C10	1.463 (3)
N1—C9	1.340 (3)	C9—H9	0.94 (2)
N1—N2	1.363 (3)	C10—H10	1.00 (3)
N1—C11	1.431 (3)	C11—C16	1.387 (3)
N2—C7	1.337 (3)	C11—C12	1.389 (3)
C1—C2	1.390 (3)	C12—C13	1.378 (3)
C1—C6	1.417 (3)	C12—H12	0.92 (2)
C2—C3	1.381 (3)	C13—C14	1.382 (3)
C3—C4	1.382 (3)	C13—H13	0.95 (2)
C3—H3	0.94 (2)	C14—C15	1.392 (3)
C4—C5	1.375 (3)	C14—H14	0.98 (2)
C4—H4	0.94 (2)	C15—C16	1.383 (3)
C5—C6	1.400 (3)	C15—H15	0.97 (2)
C5—H5	0.95 (2)	C16—H16	0.96 (2)
			
C1—O1—H1	109 (2)	C9—C8—C10	119.3 (2)
C9—N1—N2	110.51 (19)	C7—C8—C10	136.3 (2)
C9—N1—C11	129.3 (2)	N1—C9—C8	108.9 (2)
N2—N1—C11	120.21 (17)	N1—C9—H9	122.7 (15)
C7—N2—N1	106.97 (18)	C8—C9—H9	128.2 (15)
O1—C1—C2	117.8 (2)	O2—C10—C8	128.1 (3)
O1—C1—C6	123.4 (2)	O2—C10—H10	121.6 (16)
C2—C1—C6	118.8 (2)	C8—C10—H10	110.3 (16)
C3—C2—C1	122.1 (2)	C16—C11—C12	120.8 (2)
C3—C2—Cl1	118.92 (19)	C16—C11—N1	119.7 (2)
C1—C2—Cl1	118.95 (18)	C12—C11—N1	119.5 (2)
C2—C3—C4	118.8 (2)	C13—C12—C11	119.0 (2)
C2—C3—H3	119.8 (14)	C13—C12—H12	123.6 (15)
C4—C3—H3	121.3 (14)	C11—C12—H12	117.3 (15)
C5—C4—C3	120.5 (2)	C12—C13—C14	121.2 (2)
C5—C4—H4	120.4 (15)	C12—C13—H13	118.1 (15)
C3—C4—H4	119.1 (15)	C14—C13—H13	120.6 (15)
C4—C5—C6	121.5 (2)	C13—C14—C15	119.2 (2)
C4—C5—H5	120.7 (14)	C13—C14—H14	118.4 (14)
C6—C5—H5	117.8 (14)	C15—C14—H14	122.4 (14)
C5—C6—C1	118.1 (2)	C16—C15—C14	120.5 (2)
C5—C6—C7	121.1 (2)	C16—C15—H15	120.4 (14)
C1—C6—C7	120.8 (2)	C14—C15—H15	119.0 (14)
N2—C7—C8	109.3 (2)	C15—C16—C11	119.3 (2)
N2—C7—C6	118.3 (2)	C15—C16—H16	120.0 (14)
C8—C7—C6	132.4 (2)	C11—C16—H16	120.7 (14)
C9—C8—C7	104.3 (2)		

### Hydrogen-bond geometry (Å, °)

**Table:**

*D*—H···*A*	*D*—H	H···*A*	*D*···*A*	*D*—H···*A*
O1—H1···N2	0.79 (3)	1.89 (3)	2.585 (2)	147 (3)
C5—H5···O2	0.95 (2)	2.18 (2)	3.024 (3)	148 (2)
C10—H10···O1^i^	1.00 (3)	2.58 (3)	3.568 (3)	171 (2)

Symmetry codes: (i) −*x*+1, *y*−1/2, −*z*+1/2.
